# Role of RGMc as a Neogenin Ligand in Follicular Development in the Ovary

**DOI:** 10.3390/biomedicines9030280

**Published:** 2021-03-10

**Authors:** Yu Jin Kim, YoungJoon Park, Yeo Reum Park, Young Sang Kim, Hye Ran Lee, Sang Jin Lee, Myung Joo Kim, KyuBum Kwack, Jung Jae Ko, Jae Ho Lee

**Affiliations:** 1 CHA Fertility Center Seoul Station, Seoul 04637, Korea; yj_kim@chamc.co.kr (Y.J.K.); summereum@chamc.co.kr (Y.R.P.); p0892@chamc.co.kr (Y.S.K.); hrlee87@chamc.co.kr (H.R.L.); 2 Department of Biomedical Science, CHA University, Gyeonggi-do, Seongnam 13488, Korea; yjparkcb@gmail.com; 3 Institute of Animal Genetic Resources Affiliated with Traditional Hanwoo Co., Ltd., Boryeong 33402, Korea; leesj914215@naver.com

**Keywords:** ovary, neogenin, RGMc, follicular development, *PGD2*, poor ovarian response

## Abstract

There is currently no cure for infertility in women with a poor ovarian response (POR). Neogenin is reported to be abundantly expressed in the ovary; however, its role in mammalian follicular development is unclear and its ligand and signaling pathway remain uncertain. We systematically investigated the role of neogenin and the ligand repulsive guidance molecule c (RGMc) during follicular development. We treated hyperstimulated mouse ovaries with RGMc and analyzed follicular development. Furthermore, we investigated clusters of up/downregulated genes in RGMc-treated ovaries using whole-transcriptome next-generation sequencing (NGS). In addition, we investigated whether expression of up/downregulated factors identified by NGS was also altered in cumulus cells (CCs) of patients with a POR. The number of oocytes was 40% higher in RGMc-treated ovaries than in control ovaries. NGS data indicated that prostaglandin D2 (PGD2) was involved in the RGMc signaling pathway during follicular development. RGMc treatment significantly elevated the PGD2 level in culture medium of CCs obtained from patients with a POR. Our results demonstrate that RGMc as neogenin ligand promotes follicular development in ovaries via the PGD2 signaling pathway. Therefore, it may be possible to use RGMc for ovarian stimulation in patients with a POR.

## 1. Introduction

Clinically, a poor ovarian response (POR) is closely related to a low pregnancy success rate and is observed in 9–24% of infertile female patients [1]. Although great efforts have been made to treat a POR, it cannot be cured or treated with assisted reproductive technologies [2]. The European Society of Human Reproduction and Embryology defined a POR according to the “Bologna Criteria”, which are advanced maternal age older than 40 years, a previous low number of collected oocytes with a controlled ovarian stimulation (COS) protocol, and a low anti-Müllerian hormone (AMH) level (0.5–1.1 ng/mL) or an abnormal ovarian reserve test with a follicle count of less than four [3]. There are several POR-specific COS protocols including nature cycle, mild stimulation, and high-dose stimulation [4]. COS is most difficult to perform in patients who do not adequately respond to induction hormones [5]. Therefore, it is critical to improve understanding of primordial follicles and follicle development and to develop specific COS protocols for patients with a POR.

Follicle development is the recruitment of primordial follicles and maturation of antral follicles for ovulation. This process involves multiple cellular complexes between oocytes and somatic cells like granulosa cells, cumulus cells [6]. It comprises two phases: the gonadotropin-independent phase and the gonadotropin-dependent phase [6]. The gonadotropin-independent phase is regulated by transforming growth factor β family members including bone morphogenetic proteins, growth differentiation factors, activin, inhibin, and AMH between the primary and secondary follicle stages [7,8]. The gonadotropin-dependent phase is regulated by two gonadotropins, namely, follicle-stimulating hormone (FSH), which stimulates estrogen production, and luteinizing hormone (LH), which triggers ovulation and development of the corpus luteum during the reproductive cycle [8]. Approaches for overcoming a POR have sought to increase the number of preantral follicles and facilitate the development of antral follicles, which occurs in the gonadotropin-dependent phase. Induction protocols in patients with a POR target the gonadotropin signaling pathway to increase the number of antral follicles. However, regulatory factors in the gonadotropin-independent and -dependent phases that can be targeted to overcome a POR remain poorly identified.

Neogenin is a member of the immunoglobulin superfamily and functions as a transmembrane receptor with various roles in many tissues such as regulation of neuronal differentiation, inflammation, and tissue regeneration [9,10,11,12]. Neogenin has three major signaling ligands: Repulsive Guidance Molecules (RGMs; RGMa, RGMb, and RGMc), netrin-1, and slit [13,14,15]. These ligands act in various processes such as neurogenesis, mammalian gland morphogenesis, and the apoptosis pathway [10,13]. Each ligand signals via a distinct pathway and has different roles depending on the tissue and organ. mRNA expression of neogenin is higher in the ovary than in any other tissue or organ in mammals [16]. Neogenin enhances expression of Oct3/4, Nanog, and Sox2 in preimplantation embryos treated with RGMc [16,17]. However, the role of neogenin in follicular development in the ovary is completely unknown [16]. We hypothesized that neogenin controls ovarian physiology.

This study aimed to elucidate the role of neogenin in follicular development by treating hormone (pregnant mare serum gonadotropin (PMSG))-stimulated mouse ovaries with RGMc. We investigated the mechanism by which neogenin and RGMc function in follicular development by performing whole-transcriptome next-generation sequencing (NGS). Finally, we validated the identified neogenin signaling molecules using RGMc-treated cumulus cells (CCs) of patients with a POR to facilitate the development of therapeutic protocols based on the whole transcriptome.

## 2. Materials and Methods

### 2.1. Animals

Outbred white ICR mice (6–8 weeks old, 20–25 g in weight) were purchased from Oriental Bio (Seongnam, Korea). All procedures for animal breeding and care complied with the regulations of the Institutional Animal Care and Use Committee of CHA University (IACUC No. IACUC200175, 20 October 2020) (Seoul, Korea). In total, 60 mice were used for the experiment (control = 30, test =30).

### 2.2. Determination of the Effect of RGMc on Ovaries In Vivo

For in vivo hormone stimulation, the test group of mice were injected with 0.5 mg/kg RGMc and then with 75 IU PMSG 12 h later. The control was only 75IU PMSG injected group. Mice were euthanized by inhalation of CO_2_ gas in a chamber. Both ovaries were collected, and one was fixed with 4% paraformaldehyde for histological analysis. The other ovary was stored at −80 °C for analysis by whole-transcriptome NGS, RT-PCR, and Western blotting.

### 2.3. Histological Analysis and Confocal Imaging

Mouse ovaries were imaged using light and confocal microscopy. Ovaries were harvested and fixed with 4% paraformaldehyde for overnight at room temperature. Hematoxylin and eosin (H&E) staining of ovary was performed using routine protocols. We performed H&E stains with the first cross-section and the 50th cross-section of each sample ovary for counting the number of follicles’ development. For immunofluorescence staining, tissues were deparaffinized by incubation with a xylene step, and rehydrated by incubation with a graded ethanol series at room temperature. Slides were costained with a rabbit anti-neogenin antibody (1:100 diluted, H-175; Santa Cruz Biotechnology, Dallas, TX, USA) and a mouse anti-P63 antibody (1:100 diluted, D-9, Santa Cruz Biotechnology, Dallas, TX, USA). Thereafter, slides were incubated with Alexa Fluor 488- and Alexa Fluor 594-conjugated secondary antibodies (1:200 diluted, Molecular Probes, Eugene, OR, USA). Finally, samples were counterstained with Hoechst 33,342 (H1399; Thermo Fisher Scientific™, Waltham, MA, USA) for 5 min at room temperature, washed with PBS, and mounted on cover glasses with anti-fade mounting media (VECTASHIELD H-1000, Burlingame, CA, USA). Confocal microscopic images were acquired using an LSM880 microscope equipped with an Airyscan META device (Carl Zeiss, Oberkochen, Germany).

Oocytes were fixed with 4% paraformaldehyde for 10 min at room temperature. The samples were then treated with 0.1% Triton-X for antigen retrieval and were washed with Phosphate-Buffered Saline (PBS; 14,190,144, pH7.4 Thermo Fisher Life Technologies, San Diego, CA, USA) at room temperature for 30 min. A rabbit anti-neogenin antibody (1:100 diluted in PBS with 0.1% bovine serum albumin; BSA) was added, followed by incubation overnight at 4 °C. Thereafter, the samples were incubated with the secondary antibody (anti-rabbit labeled 555) for 2 h at room temperature. The oocytes were washed with PBS. The nuclei were stained with 1 µg/mL Hoechst^®^ 33342 nuclear stain (H1399; Thermo Fisher Life Technologies, San Diego, CA, USA) for 5 min at room temperature. After washing twice with PBS, the stained oocytes were transferred into a micro-drop of PBS on a glass bottom dish. Images of the stained oocytes were acquired using a confocal microscope (LSM880 with Airyscan META; Carl Zeiss AG, Oberkochen, Germany), and images were analyzed using ZEN2012 software (version 8.0, Carl Zeiss, Oberkochen, Germany).

### 2.4. Gene Expression Analysis of RGMc-Treated Ovaries

Total RNA was isolated from ovaries using TRIzol according to the manufacturer’s procedures (15596026; Invitrogen, Waltham, MA, USA). The isolated 100 ng total RNA was transcribed into cDNA using AccuPower^®^ CycleScript RT PreMix (Bioneer, Daejeon, Korea) and then amplified with AccuPower^®^ Taq PCR PreMix (Bioneer) using primer sets specific for mouse neogenin, Oct3/4, Nanog, p63, and β-actin (Table S1). In total, 10 pmol/µL forward and reverse primers and 200 ng template cDNA were added to AccuPower^®^ Taq PCR PreMix tubes, and then distilled water was added up to a total volume of 20 µL. The PCR cycling conditions were as follows: 1 min at 95 °C, followed by 32 cycles of denaturation for 30 s at 95 °C, annealing for 30 s at 60 °C, and extension for 50 s at 72 °C. PCR products were resolved on 2% agarose gels with Safeview™ (Applied Biological Materials, Richmond, BC, Canada). The gels were visualized under ultraviolet illumination using a gel documentation system (WSE-6100 LuminoGraph; ATTO, Tokyo, Japan).

### 2.5. Protein Expression Analysis of RGMc-Treated Ovaries

Protein expression in RGMc-treated ovaries was analyzed by Western blotting. Ovarian tissue samples were homogenized in PRO-PREP™ Protein Extraction Solution (iNtRON Biotechnology, Seoul, Korea). Proteins were denatured at 95 °C for 5 min in Laemmli buffer, separated by 10% sodium dodecyl sulfate polyacrylamide gel electrophoresis, and transferred onto 0.2 µm nitrocellulose membranes (Bio-Rad, Hercules, CA, USA). The membranes were blocked in TBST buffer containing 5% BSA for 1 h at room temperature and then incubated with rabbit anti-neogenin polyclonal (NBP1-89651, 1:2000; Novus, Centennial, CO, USA), rabbit anti-Oct3/4 (sc-9081, 1:2000; Santa Cruz Biotechnology, Dallas, TX, US), mouse anti-Nanog (sc-134218, 1:2000; Santa Cruz Biotechnology), mouse anti-p63 (sc-25268, 1:2000; Santa Cruz Biotechnology, Dallas, TX, USA), and mouse anti-β-actin (MA5-15739, 1:2000; Invitrogen, Waltham, MA, US) primary antibodies diluted in TBST buffer containing 1% BSA overnight at 4 °C. After washing with TBST buffer, the membranes were incubated with horseradish peroxidase-conjugated donkey anti-mouse and anti-rabbit IgG secondary antibodies (5178-2504 and 5196-2504; Bio-Rad). Immunoreactive bands were visualized using enhanced chemiluminescence (Clarity™ Western ECL Substrate 1,705,060, Bio-Rad). Images of the bands were acquired using a gel documentation system (WSE-6100 LuminoGraph I, ATTO).

### 2.6. Whole-Transcriptome NGS Analysis Comparing Control and RGMc-Treated Ovaries

The whole transcriptomes of RGMc-treated and control ovaries were analyzed by NGS. Total RNA was isolated from ovaries using TRIzol following the manufacturer’s protocols (15,596,026; Invitrogen, Waltham, MA, USA). Purified mRNA was used for NGS after its quality had been checked. Sample sheets were prepared on the MiSeq sequencer (Illumina, San Diego, CA, USA) to provide run details. NGS data were analyzed using the REVIGO program and Gene Ontology.

Differentially expressed gene (DEG) and enrichment analyses of RGMc-treated and control ovaries were performed using four *Mus musculus* samples per group. The raw sequencing data were mapped, and potential transcripts were assembled using HISAT2 (ver. 2.1.0), Bowtie2 (ver. 2.3.4.1), and StringTie (ver. 1.3.4 d). The aligned read counts were normalized with the Trimmed Mean of M-values method in edgeR. Genes whose expression exhibited an absolute fold-change of 4 or higher were extracted and clustered by an agglomerative method with Euclidean and Ward methods.

A biological process database comprising 20,196 reference genes was used for gene enrichment analyses. The ClueGO (ver. 2.5.5) module of Cytoscape (ver. 3.7.2) was used to analyze pathway enrichment for each up/downregulated gene in RGMc-treated ovaries. Significance in enrichment analysis was determined using a two-sided hypergeometric test with Bonferroni step-down corrections. A term with an enrichment percentage higher than 60% was selected for each up/downregulated gene cluster. The kappa score was calculated to identify associations between enriched terms.

### 2.7. RT-qPCR to Verify Gene Expression Changes in Ovaries

RT-qPCR was performed to confirm the NGS data obtained for control and RGMc-treated ovaries. Based on the KEGG pathway, primers targeting upregulated (*Ptgs1*, *Edn2*, and *Hpgds*) and downregulated (*Tbxa2r*, *Oxtr*, and *Adra1d*) genes were selected (Table S1). RT-qPCR was performed with total RNA isolated from ovaries using TRIzol. Total RNA was reverse-transcribed into cDNA using AccuPower^®^ CycleScript RT PreMix (Bioneer). qPCR was performed using SsoAdvanced Universal SYBR Green Supermix (#1725270, Bio-Rad) on a spectrofluorometric thermal cycler (CFX96 Touch Real-Time PCR Detection System, Bio-Rad). The reaction contained 10 pmol/µL forward and reverse primers, 200 ng template cDNA, and 2× SsoAdvanced Universal SYBR Green Supermix. Distilled water was added up to a final volume of 20 µL. The PCR cycling conditions were as follows: 3 min at 95 °C, followed by 40 cycles of denaturation for 10 s at 95 °C, annealing for 30 s at 60 °C, and extension for 20 s at 72 °C. Expression of each examined gene was normalized to β-actin. Triplicate samples were tested.

### 2.8. Validation Study Using Human CCs and Follicular Fluid

NGS data indicated that prostaglandins were key elements in the effects of RGMc on follicular development. Therefore, the relationship of prostaglandins with a POR was investigated using human follicular fluid and CCs (10 samples). Follicular fluid was collected during ovum pick-up and CCs were collected after denuding of oocytes. The collection of human material was approved by the Institutional Research and Ethical Committees of CHA University (approval number: 1044308-201611-BR-027-04, 29 June 2020). An enzyme-linked immunosorbent assay (ELISA) was performed using follicular fluid collected from patients with a normal ovarian response and patients with a POR, which was characterized by pick-up of fewer than four oocytes and an AMH level ≤1 ng/mL. The concentration of prostaglandin D2 (PGD2) (sensitivity: 9.38 pg/mL, no significant cross-reactivity or interference between PGD2 and analogues was observed. E4718; Biovision, Milpitas, CA, USA) in follicular fluid was determined with an ELISA kit (Abcam, Cambridge, UK) following the manufacturer’s protocol. Signals were read with a microplate reader (Epoch microplate spectrophotometer, BioTek Instruments, Inc., Winooski, VT, USA). Gene expression in CCs was analyzed by real-time PCR. Each sample mRNA was isolated from CCs of patients with a normal ovarian response and patients with a POR using a DynaBeads mRNA DIRECT Kit (Life Technologies, Oslo, Norway). mRNA was quantified using a NanoDrop ND-1000 spectrophotometer (Nyxor Biotech, Paris, France). Total RNA was reverse-transcribed into cDNA using AccuPower^®^ CycleScript RT PreMix (Bioneer) with poly-dT. In total, 10 pmol/µL forward and reverse primers and 200 ng template cDNA were added to AccuPower^®^ Taq PCR PreMix tubes and then distilled water was added up to a total volume of 20 µL. The human primer sets are mentioned in Table S2. The PCR cycling conditions were as follows: 1 min at 95 °C, followed by 32 cycles of denaturation for 30 s at 95 °C, annealing for 30 s at 58 °C, and extension for 50 s at 72 °C. PCR products were resolved on 2% agarose gels with Safeview™ (Applied Biological Materials). The gels were visualized under ultraviolet illumination using a gel documentation system (WSE-6100 LuminoGraph; ATTO).

### 2.9. RGMc Treatment of CCs Obtained from Patients with a POR

To investigate the effect of RGMc on CCs, samples were collected from eight control patients and eight patients with a POR. The latter had been diagnosed with infertility due to a POR according to the Bologna criteria, while the former had a normal ovarian response and normal ovarian function. All patients underwent conventional COS protocols with a gonadotropin-releasing hormone antagonist during in vitro fertilization (IVF) procedures. CCs were seeded into a 96-well plate at a density of 10^4^ cells/well in QCL media. The next day, the culture medium was replaced with that containing 50 ng/mL RGMc. After 1 day, the culture medium was harvested and measured the concentration of PGD2 in the media using ELISA kits.

### 2.10. Statistical Analysis

All data are expressed as the means ± standard error of the mean (SEM) of triplicate measurements. Statistical analyses were carried out using a one-way ANOVA analysis with Bonferroni test of variance. The significance level was set at * *p* < 0.05, ** *p* < 0.01, and *** *p* < 0.001. Significant differences are indicated by asterisks in each figure.

## 3. Results

### 3.1. Neogenin Expression in Mouse Ovaries and Oocytes

**Figure 1:**
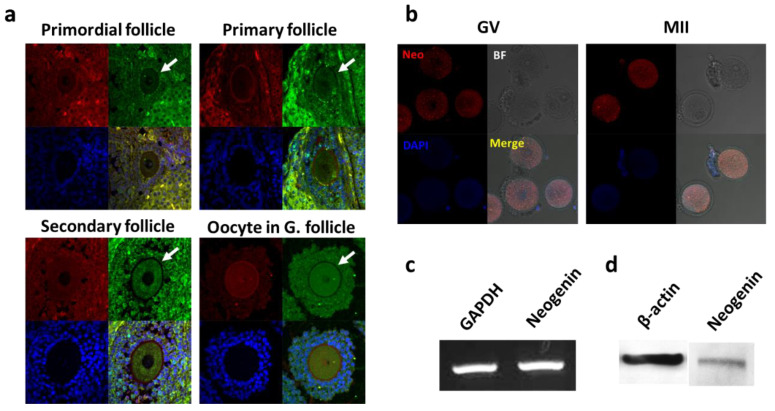
Expression profiling of neogenin in the ovary. (**a**) Confocal microscopic images of immunofluorescence staining with an anti-neogenin antibody in primordial follicle, primary follicle, secondary follicle, and oocyte in graafian follicle (oocyte in G. follicle). Neogenin, p63, and DAPI staining is shown in green, red, and blue, respectively. The arrow indicates a follicle positively stained for neogenin. (**b**) Confocal microscopic images of mouse oocytes. Neogenin and DAPI staining is shown in red and blue, respectively. GV, germinal vesicle; MII, metaphase II; BF, bright field. (**c**) RT-PCR analysis of neogenin mRNA in mouse oocytes. GAPDH was used as an internal control. (**d**) Western blot analysis of neogenin protein in mouse ovaries. β-actin was used as an internal control. Neo: Neogenin. Scale bar = 100 µm. All data are representative of three replicates.

Expression of neogenin in mouse ovaries and oocytes was examined by immunofluorescence staining. Neogenin was expressed at all stages of follicular development (Figure 1a). Staining with an anti-neogenin antibody (red) was detected in immature oocytes at the germinal vesicle and metaphase II stages (Figure 1b). Neogenin was expressed at the mRNA (Figure 1c) and protein (Figure 1d) levels in mouse ovaries.

### 3.2. RGMc Treatment Enhances Follicular Development in Hormone-Stimulated Mouse Ovaries

**Figure 2:**
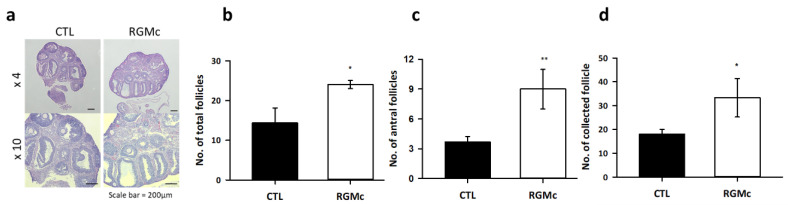
Characterization of RGMc-treated ovaries. (**a**) Light microscopic images of hematoxylin and eosin-stained mouse ovarian tissue treated without (CTL) and with RGMc. Scale bar = 200 µm. (**b**–**d**) Graphs of the total number of follicles (**b**), number of antral follicles (**c**), and number of collected oocytes (**d**) in control (CTL) and RGMc-treated ovaries. Data are the mean ± SEM of three replicates. * *p* < 0.05 and ** *p* < 0.005 versus the control group.

We evaluated the effect of RGMc treatment on mouse ovaries stimulated with PMSG in vivo. Figure 2a shows histological images of mouse ovarian tissues stained with hematoxylin and eosin following RGMc treatment and hormone stimulation. The number of total follicles, including preantral and antral follicles, was significantly higher in RGMc-treated ovaries than in control ovaries (Figure 2b). The number of antral follicles was 2.5-fold higher in RGMc-treated ovaries than in control ovaries (Figure 2c). Consequently, twice as many oocytes were collected from RGMc-treated ovaries than from control ovaries (Figure 2d). In summary, treatment with the neogenin ligand RGMc enhanced follicular development in the ovary.

### 3.3. Expression of the Primordial Follicle Factors Oct3/4, Nanog, p63, and Neogenin Is Increased in RGMc-Treated Mouse Ovaries

**Figure 3:**
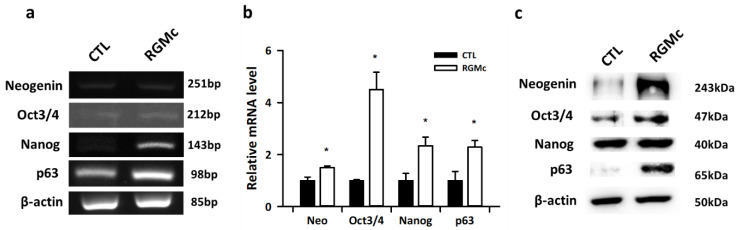
mRNA and protein expression in RGMc-treated ovaries. (**a**) RT-PCR analysis of neogenin (Neo), Oct3/4, Nanog, and p63 expression in control (CTL) and RGMc-treated ovaries. (**b**) Quantitative real-time PCR analysis of relative expression of neogenin (Neo), Oct3/4, Nanog, and p63 in control (CTL) and RGMc-treated ovaries. (**c**) Western blot analysis of neogenin (Neo), Oct3/4, Nanog, and p63 expression in control (CTL) and RGMc-treated ovaries. β-actin was used as an internal control. Data are the mean ± SEM of three replicates. * *p* < 0.05 versus the control group.

We analyzed expression of the primordial follicle factors Oct3/4, Nanog, p63, and neogenin in RGMc-treated mouse ovaries by RT-PCR. mRNA expression of Oct3/4, Nanog, p63, and neogenin was significantly higher in RGMc-treated ovaries than in control ovaries (Figure 3a). In particular, mRNA expression of Oct3/4, Nanog, and p63 was 2-fold higher in RGMc-treated ovaries than in control ovaries (Figure 3b). Protein expression of Oct3/4, Nanog, p63, and neogenin was significantly higher in RGMc-treated ovaries than in control ovaries (Figure 3c).

### 3.4. Whole-Transcriptome and Gene Enrichment Analyses of the Effects of RGMc Treatment on Hormone-Stimulated Mouse Ovaries

**Figure 4:**
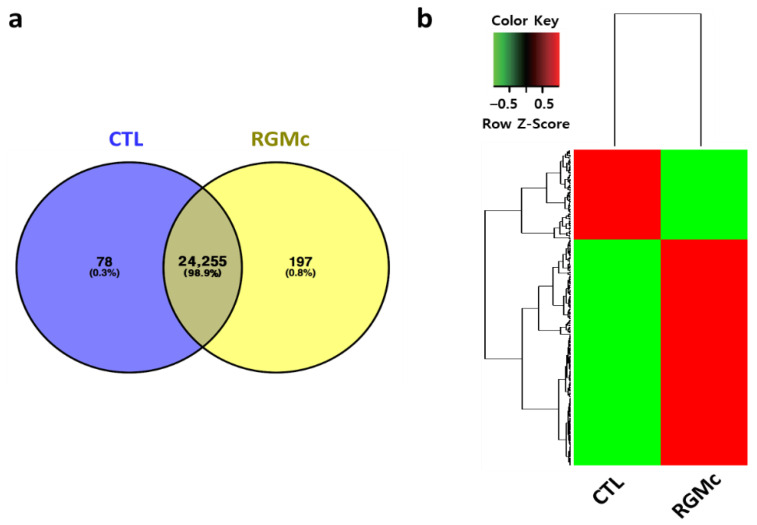
Whole-transcriptome NGS of RGMc-treated ovaries. (**a**) Venn diagram of up/downregulated genes (fold-change > 4) in control and RGMc-treated ovaries. A total of 24,255 genes were included in RNA-sequencing analysis. (**b**) Cluster map of up/downregulated genes (fold-change > 4).

Transcriptomic differences between RGMc-treated and control ovaries were investigated by RNA-sequencing. mRNA expression of 275 genes differed by more than 4-fold between RGMc-treated and control ovaries. Among these DEGs, 197 and 78 DEGs were upregulated in RGMc-treated and control ovaries, respectively (Figure 4a,b).

**Figure 5:**
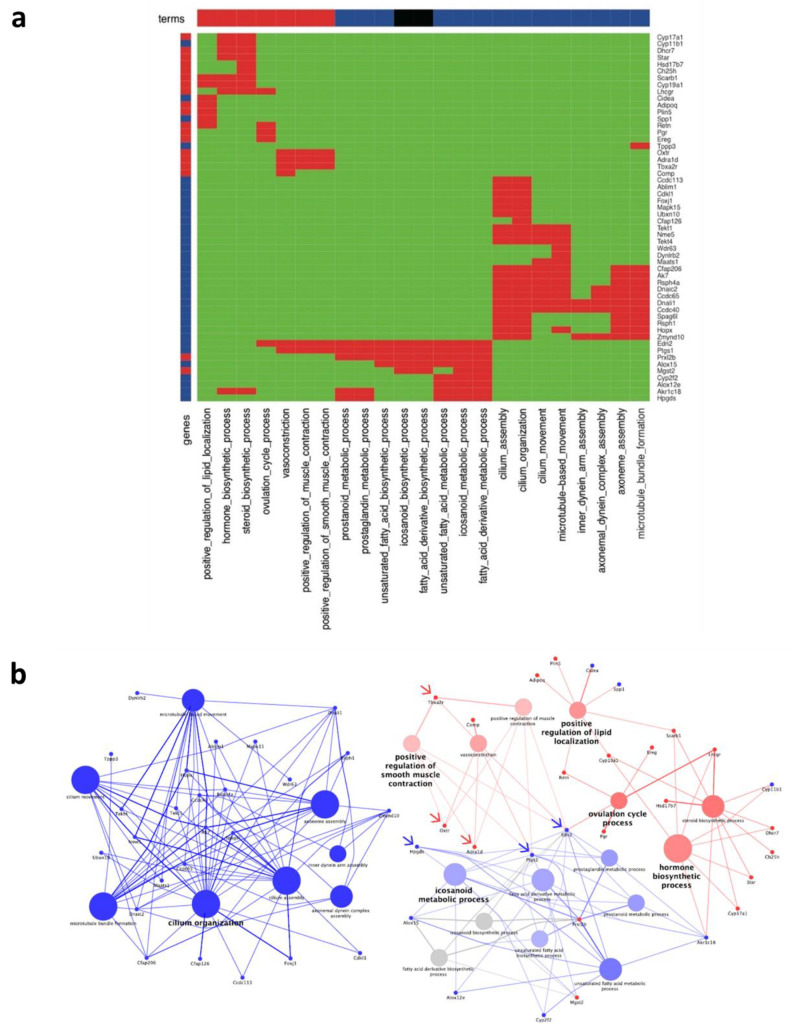
Up/downregulated genes based on an absolute fold-change of more than 4 in RGMc-treated ovaries. (**a**) Heatmap based on a binary code for significantly enriched terms and up/downregulated genes in RGMc-treated ovaries. Blue and red in the top and side bars represent upregulated and downregulated terms in RGMc-treated ovaries, respectively. Black in the top and side bars represents nonsatisfied terms with higher than 60% enrichment for up/downregulated genes. Red and green in the heatmap represent the presence and absence of the gene, respectively. (**b**) Network between significantly enriched terms and up/downregulated genes (fold-change > 4). Blue and red represent upregulated and downregulated genes/terms in RGMc-treated ovaries, respectively. Gray represents nonsatisfied terms with higher than 60% enrichment for up/downregulated genes. Blue and red arrows indicate target genes for validation that were upregulated and downregulated upon RGMc treatment, respectively.

To elucidate the mechanisms underlying hyperovulation in RGMc-treated ovaries, gene enrichment analysis of these 197 and 78 DEGs was performed. Enriched terms were determined using the ClueGO module of Cytoscape. To identify the significance of enrichment, a hypergeometric test with Bonferroni step-down corrections was applied, and 23 terms were selected. Among the 275 DEGs, 54 DEGs, including 35 upregulated and 19 downregulated DEGs, were annotated to the 23 terms (Figure 5a, Figure S1, Table S3). Protein–protein interactions for these terms were determined by Gene Ontology analysis. To determine the key genes, up/downregulated DEGs associated with the terms were plotted together using the ClueGO and CluePedia modules of Cytoscape (Figure 5b). Thicker lines represent relationships based on experimental evidence. Thinner lines represent relationships based on all other types of evidence. The size and color of circles represent the significance of enrichment and the percentage of up/downregulated genes, respectively. Among the 35 upregulated DEGs, 23 were annotated to eight cilium- and microtubule-related terms (cilium organization, cilium organization, cilium assembly, microtubule-based movement, microtubule bundle formation, axoneme assembly, axonemal dynein complex assembly, and inner dynein assembly) and were closely interconnected (Figure 5b left). The remaining 12 upregulated DEGs and all 19 downregulated DEGs were annotated to seven downregulated, six upregulated, and two nonsatisfied terms (Figure 5b right).

### 3.5. Experimental Validation of Changes in Expression of Prostaglandin and Muscle Contraction-Related Genes upon RGMc Treatment in Mice

**Figure 6:**
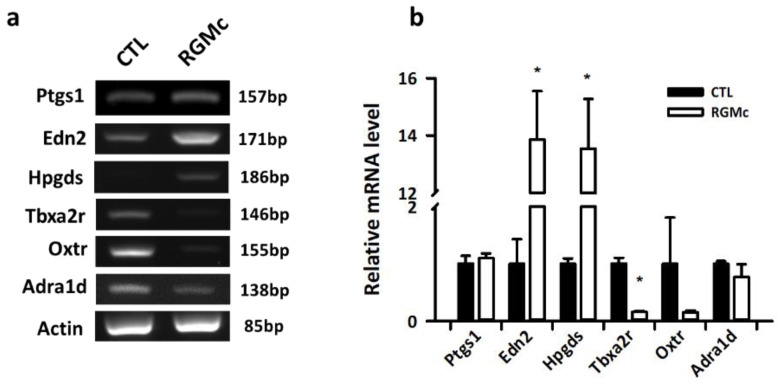
Quantitative real-time PCR analyses to validate the NGS data in RGMc-treated ovaries. (**a**) RT-PCR analysis of *Ptgs1*, *Edn2*, *Hpgds*, *Tbxa2r*, *Oxtr*, *Adra1d*, and *β-actin* expression in control (CTL) and RGMc-treated ovaries. (**b**) Quantitative real-time PCR analysis of *Ptgs1*, *Edn2*, *Hpgds*, *Tbxa2r*, *Oxtr*, and *Adra1d* expression in control (CTL) and RGMc-treated ovaries normalized against *β-actin* expression. * *p* < 0.05 versus the control group.

After performing transcriptomic analyses, we experimentally validated the changes in mRNA expression of three upregulated (Figure 5b, blue arrows) and three downregulated (Figure 5b, red arrows) genes upon RGMc treatment by real-time qPCR (Figure 6a). Among these genes, mRNA expression of hematopoietic prostaglandin D synthase (*Hpgds*) and endothelin 2 (*Edn2*) was more than 13-fold significantly higher in RGMc-treated ovaries than in control ovaries (*p* < 0.05), while mRNA expression of a downregulated gene, namely, thromboxane A2 receptor (*Tbxa2r*), was significantly decreased in RGMc-treated ovaries (*p* < 0.05) (Figure 6b). In addition, although not statistically significant, oxytocin receptor (*Oxtr*) was also markedly downregulated in RGMc-treated ovaries (Figure 6a,b).

### 3.6. The Level of PGD2 in Follicular Fluid and Culture Medium of RGMc-Treated CCs Obtained from Patients with a POR

**Figure 7:**
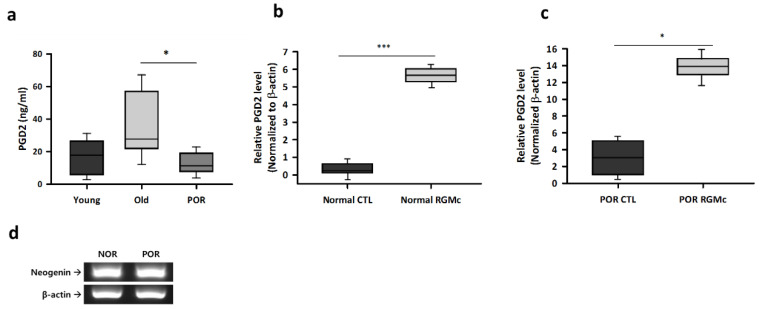
An ELISA of PGD2 in human follicular fluid and the culture medium of RGMc-treated CCs. (**a**) The PGD2 level in follicular fluid of patients with a POR and young and old patients with a normal ovarian response. (**b**,**c**) The ELISA data of PGD2 level in the culture media of RGMc-treated CCs obtained from (**b**) patients with a normal ovarian response and (**c**) patients with a POR. (**d**) Gene expression of neogenin in human CCs of normal and POR patients. All assays were repeated three times with different samples for statistical analysis. * *p* < 0.05 and *** *p* < 0.001 versus the young and old normal ovarian response groups.

We analyzed the level of PGD2 in the follicular fluid of patients with a normal ovarian response and patients with a POR using an ELISA. The PGD2 concentration was significantly lower in the follicular fluid of patients with a POR than in the follicular fluid of young and old patients with a normal ovarian response (Figure 7a). Next, we confirmed that CCs of normal and POR patients expressed neogenin and therefore analyzed the effect of RGMc treatment on CCs obtained from patients with a normal ovarian response and patients with a POR (Figure 7d). Treatment with RGMc significantly increased the PGD2 level in the culture medium of CCs obtained from patients with a POR by 4-fold (Figure 7c) and also dramatically increased the PGD2 level in the culture medium of CCs obtained from patients with a normal ovarian response (Figure 7b).

## 4. Discussion

In this study, we found that neogenin and the ligand RGMc are involved in both the gonadotropin-independent and -dependent phases of follicular development. Neogenin is expressed at various stages of follicular development in the ovary from primordial follicles to graafian follicles. Thus, RGMc treatment enhanced both the gonadotrophin-independent and -dependent phases upon hormone hyperstimulation. Our results demonstrated that RGMc treatment enhanced the activity of primordial follicles. Oct3/4, Nanog, and p63 are markers of primordial follicles and regulate the proliferation of stromal cells and their differentiation of granulosa cells supplement into preantral follicles development [18]. RGMc may facilitate COS by upregulating Oct3/4 and Nanog, thereby enhancing survival of primordial follicles in the ovary. p63 is a regulator of meiosis and is important for cell cycle control in primordial follicles [19]. RGMc treatment promoted follicular development in the ovary. In particular, it upregulated prostaglandins including PGD2. Prostaglandins regulate both the gonadotrophin-independent and -dependent phases in the ovary. In summary, RGMc promoted follicular development by binding to neogenin and upregulating PGD2.

We investigated the mechanism by which RGMc treatment enhances follicular development in the ovary by performing whole-transcriptome NGS. This revealed that 197 and 78 genes were upregulated and downregulated in RGMc-treated ovaries, respectively. We determined which terms were significantly enriched among the upregulated genes (fold-change > 4). RGMc-treated ovaries exhibited upregulation of genes involved in microtubule formation and cilia organization [20,21,22]. Microtubules in granulosa cells have been linked with cholesterol movement for steroidogenesis in mitochondria upon FSH stimulation [22]. Cilia function in steroidogenesis to produce various molecules, such as estrogen in granulosa cells via Ift88 [20]. Mice with conditional knockdown of Ift88 exhibit abnormal estrogen production in the ovaries and defects in ovarian function. A series of adenylate kinases including AK7 is translated in germinal vesicle-stage oocytes and linked with cilia function for steroidogenesis [23]. RGMc treatment upregulated *Edn2* and genes involved in microtubule bundle formation. There are three endothelin proteins called EDN1, EDN2, and EDN3. These vasoactive peptides contain 21 amino acids and reportedly function in various processes in the ovary, including steroidogenesis, follicles development, and ovulation [24]. During the reproductive cycle, EDN2 functions in the rupture of the follicle wall by regulating blood pressure and corpus luteum formation in the ovary [25,26]. *Edn2*-knockout mice exhibit a poor ovulatory response to superovulation induction [26]. Our data demonstrate that RGMc treatment enhanced follicular development by upregulating *Edn2*. The role of *Edn2* in ovulatory competence requires further investigation. RGMc treatment also upregulated *Hpgds* in the ovary. HPGDS produces PGD1 and PGD2, thereby interfering with the actions of FSH in granulosa cells [27]. Signaling of PGD2 produced by HPGDS controls expression of the FSH and LH receptors [27]. PGD2 modulate the balance between proliferation, differentiation, and steroidogenic activity in granulosa cells during the gonadotropin-independent and -dependent phases, and are thus important for the ovulatory cascade including oocyte maturation, cumulus expansion, and follicle maturation [28].

Downregulation of genes in RGMc-treated ovaries prevents premature ovulation and facilitates normal follicles development. One downregulated gene cluster was Oxtr signaling, which is associated with various biological functions in female reproductive organs. Oxytocin and Oxtr, which function in steroidogenesis, are associated with a POR, and their levels are elevated in the follicular fluid of women with PCOS [29,30,31]. Endogenous oxytocin might be involved in the regulation of LH in women [32]. However, the role of Oxtr is unclear, and further studies are required to elucidate its relationship with follicles development. *Tbxa2r* was also downregulated in RGMc-treated ovaries. However, *Tbxa2r* has not been investigated in the ovary, and a further study is required to determine the significance of its downregulation in reproductive organs.

In the validation study, we measured the concentrations of prostaglandins in human follicular fluid and found that PGD2 supports ovarian function and female fertility. PGD2 is produced by HPGDS in granulosa cells. It plays an important role and modulates the proliferation and differentiation of granulosa cells and steroidogenic activity in these cells. Our data demonstrated that the PGD2 level was lower in the follicular fluid of patients with a POR than in the follicular fluid of patients with a normal ovarian response. A POR was associated with prostaglandin metabolic defects. RGMc treatment may be able to overcome a POR for COS during IVF cycles. The PGD2 signaling pathway may play a key functional role in normal follicular development upon hormone stimulation during IVF cycles. We suggest that PGD2 is a potential biomarker to diagnose a POR. Treatment with RGMc dramatically increased the PGD2 level in the culture medium of CCs obtained from patients with a POR. These data show a possibility for PORs therapeutic factor which is promoted follicular development and that RGMc can be used to develop new COS protocols for personalized induction in women with a POR.

**Figure 8:**
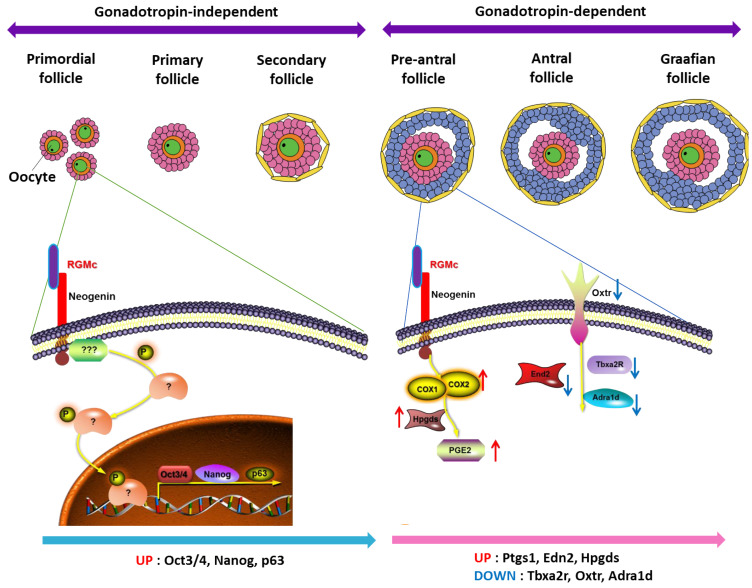
Schematic illustration of the signaling pathway by which the neogenin ligand RGMc enhances follicular development.

Taken together, RGMc affects the gonadotropin-independent and -dependent phases of follicular development via neogenin signaling. We found that neogenin in the ovary is associated with several signaling molecules including EDN2, HPGDS, and PGD2 (Figure 8). Moreover, RGMc treatment significantly increased the level of PGD2 in the culture medium of human CCs. CCs are important for follicles development and oocyte quality as supporting cells in the ovary [33,34,35], which are responsible for nurturing oocyte growth, development, and the gradual acquisition of developmental competence [36]. Therefore, the metabolic activity of CCs upon RGMc treatment may improve oocyte competence to generate good-quality preimplantation embryos.

In conclusion, neogenin has a potential role in primordial follicles and follicular development in the ovary. Despite many studies, an efficient stimulation protocol for patients with a POR is lacking. Most treatments that improve pregnancy rates have not been recommended in patients with a POR, and a POR remains one of the most challenging conditions to treat in reproductive medicine. New COS protocols incorporating RGMc should be investigated to enhance follicles development for IVF in infertile female patients with a POR.

## Supplementary Materials

The following are available online at https://www.mdpi.com/2227-9059/9/3/280/s1. Figure S1: Pairwise cluster map of kappa scores with each significant term. Table S1: Primer sequences used for real-time qPCR of RGMc-treated mouse ovaries. Table S2: Primer sequences used for qPCR of RGMc-treated human CCs. Table S3: Protein–protein interactions (PPIs) for the gene enrichment terms of DEGs determined using Gene Ontology. Of 197 upregulated (yellow and orange) and 78 downregulated (blue and green) DEGs, 35 and 19 DEGs were associated with the terms, respectively.
