# Supplementary material for: Role of RGMc as a Neogenin Ligand in Follicular Development in the Ovary
Source: Biomedicines. 2021 Mar 10;9(3):280. doi: 10.3390/biomedicines9030280 (PMC7999520; doi:10.3390/biomedicines9030280)
Supplement: Supplementary file 1 [file biomedicines-09-00280-s001.zip › biomedicines-1103248-second proof-supp/supplementary figure.docx]

**Figure S1**


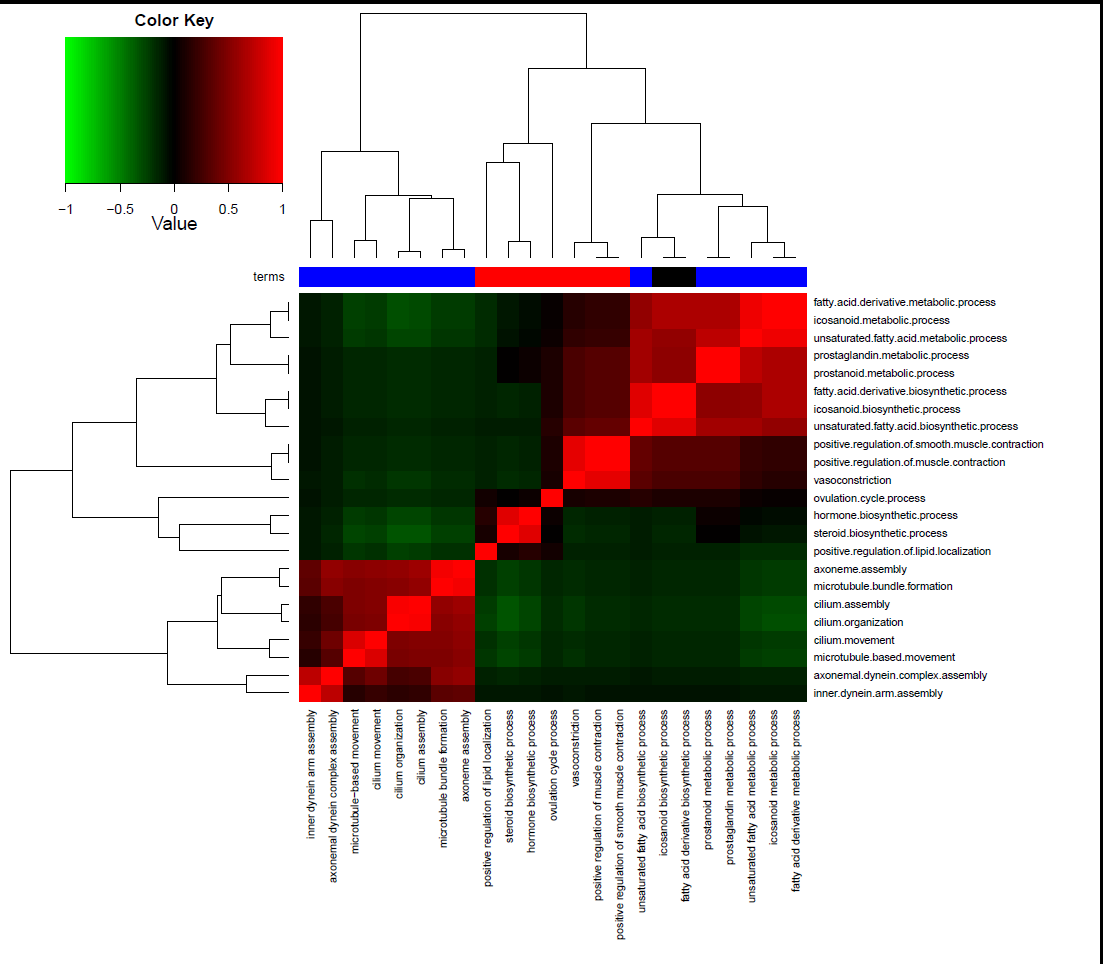


**Figure S1.** Pair-wise cluster map of kappa score with each significant term. Blue and red on column side bar represented up- and down-regulated terms in RGMc group, respectively. The black colors on column side bar represented not satisfied terms with above 60% of enrichment percentage of either up- or down-regulated genes.
