# Supplementary material for: Role of RGMc as a Neogenin Ligand in Follicular Development in the Ovary
Source: Biomedicines. 2021 Mar 10;9(3):280. doi: 10.3390/biomedicines9030280 (PMC7999520; doi:10.3390/biomedicines9030280)
Supplement: Supplementary file 1 [file biomedicines-09-00280-s001.zip › biomedicines-1103248-second proof-supp/supplementary tables.docx]

**Supplementary**

**Table S1** Primer sequences set of realtime-qPCR for RGMc treated mouse ovary

| Gene | | Primer sequences (5’-3’) | |
| --- | --- | --- | --- |
| Neogenin | Neogenin | F | GTA TGT CGC CTC GCT ACC TG |
|  |  | R | GCC ACA GAG AAG TCA TCG GA |
| Oct3/4 | Octamer-binding transcription factor 4 | F | CAC GAG TGG AAA GCA ACT CA |
|  |  | R | AGA TGG TGG TCT GGC TGA AC |
| Nanog | Nanog | F | CAC CCA CCC ATG CTA GTC TT |
|  |  | R | ACC CTC AAA CTC CTG GTC CT |
| P63 | P63 | F | GTA TCG GAC AGC GCA AAG AAC G |
|  |  | R | CTG GTA GGT ACA GCA GCT CAT C |
| Ptgs1 | Prostaglandin-endoperoxide synthase 1 | F | CCT CGA CAA CTA CCA GTG TG |
|  |  | R | CAC AAA TTC CCA GAG CCA GT |
| Edn2 | Endothelin 2 | F | GAC CTC CTC CGA AAG CTG AG |
|  |  | R | CCG TTT CCT CCT GTC TCC AC |
| Hpgds | Hematopoietic Prostaglandin D Synthase | F | GGA CAC GCT GGA TGA CTT CA |
|  |  | R | TCC CAG TAG AAG TCT GCC CA |
| Oxtr | Oxytocin Receptor | F | GTT CTC AAC CAT CCT CGG CA |
|  |  | R | CTA ACC AGC CCA AGG ACA GG |
| Tbxa2r | Thromboxane receptor | F | TCG GGC TCA TAT TCG CAC TC |
|  |  | R | AAC CAT CAT CTC CAC CTC GC |
| Adra1d | Adrenergic  receptor, alpha 1d | F | TCT TCG TCC TGT GCT GGT TC |
|  |  | R | AGA TGA GCG GGT TCA CAC AG |
| β-Actin | β-Actin | F | CAT TGC TGA CAG GAT GCA GAA GG |
|  |  | R | TGC TGG AAG GTG GAC AGT GAG G |

**Table S2** Primer sequences set of qPCR for RGMc treated human cumulus cells

| Gene | | Primer sequences (5’-3’) | |
| --- | --- | --- | --- |
| Neogenin | Neogenin | F | ATT TCC ACT CCA GCA GCC TC |
|  |  | R | GTG GAA TTG GCC CTG TCT GA |
| β-Actin | β-Actin | F | ACA ATG TGG CCG AGG ACT TT |
|  |  | R | TGT GTG GAC TTG GGA GAG GA |

**Table S3** Protein-protein interactions (PPI) among the terms of gene enrichment of DEGs were performed using Gene Ontology. Of 197 up-(yellow, orange) and 78 down-regulated DEGs(blue, green) 35 and 19 DEGs were associated with terms, respectively.

| **Gene_Symbol** | **CTR_FPKM** | **RGMC_FPKM** | **fold change Log(RGMc/CTR)** | **Description** |
| --- | --- | --- | --- | --- |
| Cyp17a1 | 306.144 | 6.863 | -5.288 | cytochrome P450, family 17, subfamily a, polypeptide 1 |
| Hsd17b7 | 212.435 | 8.201 | -4.536 | hydroxysteroid (17-beta) dehydrogenase 7 |
| Cyp19a1 | 84.397 | 3.119 | -4.374 | cytochrome P450, family 19, subfamily a, polypeptide 1 |
| **Lhcgr** | 351.147 | 24.102 | -3.810 | luteinizing hormone/choriogonadotropin receptor |
| Plin5 | 39.667 | 2.444 | -3.562 | perilipin 5 |
| Ereg | 187.549 | 15.736 | -3.494 | epiregulin |
| Comp | 35.682 | 3.598 | -2.996 | cartilage oligomeric matrix protein |
| Mgst2 | 49.875 | 5.625 | -2.941 | microsomal glutathione S-transferase 2 |
| **Oxtr** | 66.416 | 8.117 | -2.886 | oxytocin receptor |
| Pgr | 16.018 | 1.427 | -2.810 | progesterone receptor |
| Star | 1379.972 | 198.800 | -2.789 | steroidogenic acute regulatory protein |
| Ch25h | 13.133 | 1.561 | -2.464 | cholesterol 25-hydroxylase |
| Retn | 71.088 | 12.305 | -2.438 | resistin |
| Scarb1 | 1508.161 | 289.476 | -2.377 | scavenger receptor class B, member 1 |
| Dhcr7 | 206.102 | 41.704 | -2.278 | 7-dehydrocholesterol reductase |
| **Adra1d** | 7.510 | 0.806 | -2.237 | adrenergic receptor, alpha 1d |
| Prxl2b | 69.950 | 15.605 | -2.095 | family with sequence similarity 213, member B |
| Adipoq | 51.806 | 11.828 | -2.041 | adiponectin, C1Q and collagen domain containing |
| **Tbxa2r** | 6.931 | 0.963 | -2.015 | thromboxane A2 receptor |
| Ccdc65 | 0.289 | 4.224 | 2.019 | coiled-coil domain containing 65 |
| Ccdc40 | 0.126 | 3.719 | 2.068 | coiled-coil domain containing 40 |
| Wdr63 | 0.008 | 3.325 | 2.101 | WD repeat domain 63 |
| Ak7 | 0.675 | 6.502 | 2.163 | adenylate kinase 7 |
| Tppp3 | 21.249 | 99.110 | 2.170 | tubulin polymerization-promoting protein family member 3 |
| Hopx | 2.341 | 14.104 | 2.177 | HOP homeobox |
| Maats1 | 0.207 | 4.498 | 2.187 | MYCBP-associated, testis expressed 1 |
| **Hpgds** | 0.739 | 7.038 | 2.208 | hematopoietic prostaglandin D synthase |
| Ccdc113 | 0.061 | 3.918 | 2.213 | coiled-coil domain containing 113 |
| Ablim1 | 4.628 | 25.171 | 2.217 | actin-binding LIM protein 1 |
| Tekt4 | 0.121 | 4.771 | 2.365 | tektin 4 |
| **Ptgs1** | 3.535 | 22.359 | 2.365 | prostaglandin-endoperoxide synthase 1 |
| Mapk15 | 0.107 | 4.742 | 2.375 | mitogen-activated protein kinase 15 |
| Spag6l | 0.108 | 4.776 | 2.383 | sperm associated antigen 6-like |
| Cfap126 | 3.782 | 24.299 | 2.403 | cilia and flagella associated protein 126 |
| Dnaic2 | 0.471 | 7.022 | 2.447 | dynein, axonemal, intermediate chain 2 |
| Rsph4a | 0.113 | 5.083 | 2.450 | radial spoke head 4 homolog A (Chlamydomonas) |
| Cyp2f2 | 1.387 | 12.871 | 2.539 | cytochrome P450, family 2, subfamily f, polypeptide 2 |
| Zmynd10 | 0.102 | 5.441 | 2.547 | zinc finger, MYND domain containing 10 |
| Tekt1 | 0.316 | 7.113 | 2.625 | tektin 1 |
| **Edn2** | 6.280 | 47.040 | 2.722 | endothelin 2 |
| Cidea | 0.198 | 7.112 | 2.759 | cell death-inducing DNA fragmentation factor, alpha subunit-like effector A |
| Cdkl1 | 0.771 | 11.140 | 2.777 | cyclin-dependent kinase-like 1 (CDC2-related kinase) |
| Nme5 | 0.948 | 12.599 | 2.804 | NME/NM23 family member 5 |
| Rsph1 | 1.639 | 17.521 | 2.811 | radial spoke head 1 homolog (Chlamydomonas) |
| Cfap206 | 0.316 | 8.329 | 2.826 | cilia and flagella associated protein 206 |
| Dnali1 | 0.377 | 9.908 | 2.986 | dynein, axonemal, light intermediate polypeptide 1 |
| Ubxn10 | 0.205 | 8.892 | 3.038 | UBX domain protein 10 |
| Akr1c18 | 0.332 | 10.994 | 3.170 | aldo-keto reductase family 1, member C18 |
| Alox15 | 1.012 | 23.912 | 3.630 | arachidonate 15-lipoxygenase |
| Alox12e | 0.458 | 17.905 | 3.697 | arachidonate lipoxygenase, epidermal |
| Foxj1 | 0.744 | 24.676 | 3.880 | forkhead box J1 |
| Spp1 | 8.937 | 150.448 | 3.930 | secreted phosphoprotein 1 |
| Dynlrb2 | 1.345 | 35.420 | 3.957 | dynein light chain roadblock-type 2 |
| Cyp11b1 | 0.616 | 26.243 | 4.075 | cytochrome P450, family 11, subfamily b, polypeptide 1 |
